# Long-term effects of a food pattern on cardiovascular risk factors and age-related changes of muscular and cognitive function

**DOI:** 10.1097/MD.0000000000022381

**Published:** 2020-09-25

**Authors:** Charlotte Wernicke, Konstantina Apostolopoulou, Silke Hornemann, Andriana Efthymiou, Jürgen Machann, Sein Schmidt, Uwe Primessnig, Manuela M. Bergmann, Tilman Grune, Christiana Gerbracht, Katharina Herber, Anne Pohrt, Andreas F.H. Pfeiffer, Joachim Spranger, Knut Mai

**Affiliations:** a Charité - Universitätsmedizin Berlin, Corporate Member of Freie Universität Berlin, Humboldt-Universität zu Berlin, and Berlin Institute of Health, Department of Endocrinology and Metabolism, 10117 Berlin; b NutriAct-Competence Cluster Nutrition Research, Berlin-Potsdam; c Department of Clinical Nutrition, German Institute of Human Nutrition, Potsdam-Rehbruecke, Nuthetal; d Institute for Diabetes Research and Metabolic Diseases (IDM) of the Helmholtz Center Munich at the University of Tübingen, Tübingen; e German Center for Diabetes Research, München-Neuherberg; f Section on Experimental Radiology, Department of Diagnostic and Interventional Radiology, University Hospital Tübingen, Tübingen; g Charité - Universitätsmedizin Berlin, Corporate Member of Freie Universität Berlin, Humboldt-Universität zu Berlin, and Berlin Institute of Health, Clinical Research Unit, 10117 Berlin; h DZHK (German Centre for Cardiovascular Research), Partner Site Berlin, Berlin; i Department of Internal Medicine and Cardiology, Charité - Universitätsmedizin Berlin, Campus Virchow-Klinikum, Berlin; j Charité - Universitätsmedizin Berlin, Corporate Member of Freie Universität Berlin, Humboldt-Universität zu Berlin, and Berlin Institute of Health, Institute of Biometry and Clinical Epidemiology, Berlin, Germany.

**Keywords:** cardiovascular function, cognition, diet, dietary fibre, dietary proteins, healthy aging, metabolism, sarcopenia, unsaturated fatty acids

## Abstract

**Introduction:**

The mean age of the German population increased over the last years, which resulted in a higher prevalence of cardiovascular diseases, type 2 diabetes, cognitive impairment, sarcopenia and bone fractures. Current evidence indicates a preservation of human wellbeing in the elderly by a healthy diet, although the recommended macronutrient composition and quality remains unclear and needs further long-term investigation. In this context we investigate the effect of a specific dietary pattern on age-related disorders in a randomized controlled multi-center trial (RCT).

**Methods:**

We assess the effect of a specific dietary pattern (NutriAct) with a high proportion of unsaturated fat, plant proteins and fibres (fat 35%–40% of total energy (%E) of which 15%E–20%E monounsaturated fatty acids (MUFA) and 10%E–15%E polyunsaturated fatty acids (PUFA), 15%E–25%E proteins, ≥30 g fibres per day and 35%E–45%E carbohydrates) on age-related impairment of health within a 36-months RCT conducted in the region of Berlin and Potsdam. 502 eligible men (n = 183) and women (n = 319), aged 50 to 80 years, with an increased risk to develop age-related diseases were randomly assigned to either an intervention group focusing on NutriAct dietary pattern or a control group focusing on usual care and dietary recommendations in accordance to the German Nutrition Society (DGE). In the intervention group, 21 nutrition counsellings as well as supplementation of rapeseed oil, oil cake and specific designed foods are used to achieve the intended NutriAct dietary pattern.

The primary outcome is a composite endpoint of age-related disorders, including cardiovascular morbidity, decline of cognitive function as well as clinical features of sarcopenia. Secondary outcomes include diet-induced effects on quality of life, depression, frailty, cardiovascular function, bone density, fat distribution pattern, glucose, lipid and energy metabolism, as well as the identification of biomarkers linked with age-related disorders.

**Discussion:**

The findings of this trial will provide clinically relevant information regarding dietary effects on age-related impairment of health and will contribute to the definition of the optimal macronutrient composition in the context of healthy aging in the German population.

## Abstract 2

Supplemental Digital Content is available in the text

## 1 Introduction

The aging of the population is associated with a substantial rise of cardiovascular diseases (CVD), metabolic disorders like type 2 diabetes and non-alcoholic fatty liver disease, cancer, dementia and sarcopenia.^[1–4]^ The dietary pattern is thought to constitute a major contributing factor^[5,6]^ and seemed to be causally linked to 17.9% of CVD-related deaths in Germany in 2016.^[7]^ Vice versa, current evidence from predominantly epidemiological studies indicates the reduction of age-related diseases through a healthy diet.^[4,5,8]^ Strongest evidence for the power of healthy nutrition to prevent cardiovascular endpoints has been shown for the Mediterranean diet,^[9]^ which is particularly characterized by high intake of unsaturated fatty acids.

Over the last years, research has shifted from analysis of single macronutrients to the evaluation of dietary patterns, as numerous beneficial interactive effects are evident.^[10]^ However, a considerable uncertainty exists regarding the optimal macronutrient composition and quality for improving health status in the elderly. Exemplarily, the preferred amount of proteins (high protein vs low protein) is under debate.^[11,12]^ In middle-aged humans (<65 years), high protein intake was linked to an increased overall and cancer-related mortality, though this association was abolished or significantly reduced if the proteins were plant-derived.^[12]^ Accordingly, higher intake of plant protein seems to be associated with decreased cardiovascular, cancer-related and all-cause mortality compared to animal protein.^[13–15]^ Especially in the elderly, high protein intake seems to be beneficial. High protein intake was even associated with a reduced all-cause and cancer-related mortality in people older than 65 years.^[12]^ Furthermore, high consumption of dietary protein did improve left ventricular function and remodeling in middle-aged patients with type 2 diabetes^[16]^ as well as functional exercise capacity in heart failure in elderly patients >65 years.^[17]^ Moreover, observational studies also indicate a beneficial effect on maintenance of physical function^[18]^ and prevention of frailty and sarcopenia in older adults.^[4,19]^

Modulation of fat intake might also improve age-related decline in health. While liver fat and serum cholesterol are elevated by saturated fatty acids (SFA), increase of mono- (MUFA) and polyunsaturated fatty acids (PUFA) seems to be beneficial for modulation of liver fat and lipid metabolism.^[20–23]^ Diets focusing on high MUFA and PUFA intake also improved insulin sensitivity,^[24]^ risk of future type 2 diabetes^[25]^ and cardiovascular outcome,^[9,26]^ especially in a Mediterranean population. Nevertheless, the preventive role of PUFA in promoting cardiovascular health has been debated in recent meta-analyses showing little or no effect.^[27,28]^ Apart from that, adherence to the Mediterranean diet with high content of unsaturated fatty acids was associated with decreased risk of age-related cognitive decline in predominantly observational studies.^[8]^

Furthermore, a low glycemic index diet has been shown to improve glycemic control particularly in type 2 diabetes.^[29]^ It might also reduce body weight, insulin resistance and low-density lipoprotein (LDL) cholesterol in obese subjects,^[30,31]^ while increased fibre intake has been associated with reduction of cardiovascular risk and risk of type 2 diabetes.^[32,33]^

Taken together, available data suggests that a dietary pattern based on high intake of plant protein, MUFA, PUFA and fibre may improve healthy aging, even if sufficiently powered RCTs with long-term follow-up are currently not available.

## 2 Objectives

We intend to assess the effect of a dietary approach focusing on high intake of unsaturated fat, plant proteins and fibre combined with a low glycemic index (NutriAct pattern) for healthy aging in middle-aged and elderly people with increased risk for age-related disorders. Therefore, we initiated a randomized controlled 36-months dietary intervention trial comparing the NutriAct dietary pattern and usual care including dietary advices for a healthy diet based on recommendations of the German Nutrition Society (DGE).^[34]^

## 3 Methods: participants, intervention, and outcomes

### 3.1 Study design

The aim of the here described randomized controlled multi-center parallel group trial named “NutriAct” is to compare long-term effects of 2 different dietary patterns in a German aging population. Therefore, 502 eligible persons were enrolled and randomly assigned to intervention or control group after initial characterization. The description of the study design follows the guidelines of the SPIRIT 2013 Statement using the Spirit checklist (see additional file).^[35]^ The study protocol was approved by the Institutional Review Board of the Charité Medical School. The trial is conducted in accordance to the Declaration of Helsinki. All subjects gave written informed consent prior to inclusion in the study. The trial was registered at German Clinical Trials Register (DRKS00010049).

### 3.2 Study setting

The clinical trial is a multi-center study carried out at the Metabolic Research Unit of the Clinic of Endocrinology, Diabetes and Metabolism, Charité - Universitätsmedizin Berlin and the Human Study Center of the German Institute of Human Nutrition (DIfE) Potsdam-Rehbruecke.

### 3.3 Eligibility criteria

Potential participants of the study are men and women aged between 50 and 80 years with at least one risk factor for unhealthy aging as follows: elevated blood pressure (systolic blood pressure ≥140 mm Hg or diastolic blood pressure ≥90 mm Hg or medical history of hypertension or use of antihypertensive medication), known CVD (stroke, myocardial infarction, coronary heart disease (CHD), peripheral artery disease (PAD)), heart failure (defined by New York Heart Association (NYHA) ≥II or NT-Pro-BNP >300 ng/l in absence of atrial fibrillation), cognitive impairment (Montreal Cognitive Assessment (MoCA) Score <26) or decreased physical function (Short Physical Performance Battery (SPPB) Score <10).

Exclusion criteria are the presence of acute severe CVD including unstable CVD, recent cardiovascular event or surgery ≤ 3 months, type 1 diabetes mellitus, type 2 diabetes mellitus under insulin therapy, uncontrolled hypertension (blood pressure values >180 mm Hg systolic and/or 110 mm Hg diastolic), life expectancy <1 year, prevalent cancer, severe hepatic or renal diseases (estimated GFR <50 ml/min/1.73 m^2^), severe gait disturbance diseases (e.g. Parkinson's disease, stroke with paresis), severe systemic infection, severe immune disease, severe food allergy, severe malabsorption disease, oral glucocorticoid treatment, untreated active endocrine disease, severe psychiatric disorder, severe drug and/or alcohol abuse, mental limitations or known eating disorder.

### 3.4 Intervention

**Figure 1:**
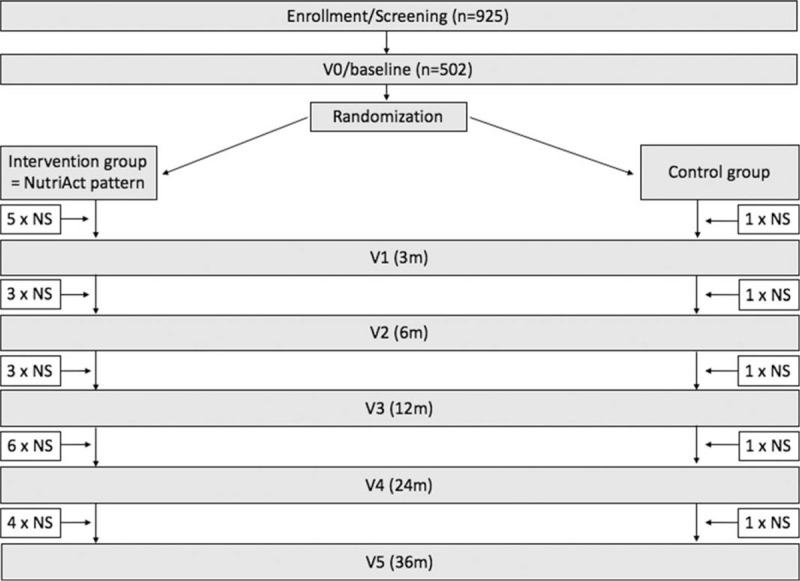
NutriAct study flowchart. The NutriAct pattern is implemented in the intervention group. The control group is a usual care group including dietary recommendations of the German Nutrition Society. Phenotyping is being performed at baseline (V0), at 3 m (V1), 6 m (V2), 12 m (V3), 24 m (V4) and 36 m (V5) after start of intervention. Sessions of nutrition counselling take place repeatedly between the visits as indicated. n = number, NS = nutrition session, m = months, V = visit.

Two different dietary patterns are compared within this RCT. The entire intervention period is 36 months. Within the intervention group a specific NutriAct dietary pattern is implemented. This pattern is defined by the composition of the macronutrients. It consists of 35% to 40% energy (%E) total fat with a high proportion of unsaturated fat, 15%E to 25%E protein emphasizing plant proteins, 35%E to 45%E carbohydrates focusing on low glycemic index and at least 30 g fibres per day. The exact composition of dietary fat is intended as follows: SFA ≤ 10%E, MUFA 15%E to 20%E und PUFA 10%E to 15%E. Therefore, SFA are replaced by MUFA and PUFA. Advice on diets and lifestyle as well as practical cookery courses are administered by professional dieticians within 21 group sessions over the entire trial (see Fig. 1 and Supplemental Digital Content (Fig. S1)). This approach is supported by supplementation of rapeseed oil instead of butter and cream. Therefore, all participants in the intervention group receive one liter of rapeseed oil per month as well as 500 g of oil cake every 6 weeks at no cost. In addition, different specific designed NutriAct foods are supplemented in the intervention group on a regular basis to modify food intake according to the NutriAct pattern. Examples are protein and fibre enriched bread rolls and pasta as well as flakes with a high protein and a low carbohydrate content. Furthermore, a high intake of vegetables and nuts as well as moderate fruit intake is being encouraged.

The control group receives usual care in accordance to local standard care based on dietary recommendations of the DGE.^[34,36]^ This includes 5 sessions of nutrition counselling held by professional dieticians according to Figure 1. Thereby, we recommend a moderate total fat (30%E; SFA ≤ 10%E, MUFA ≥ 10%E, PUFA 7%E–10%E), high carbohydrate (55%E) and moderate protein (15%E) intake.^[34,36]^ Participants of the control group intermittently receive high carbohydrate muesli, barley flakes and barley as an alternative to rice throughout the study period at no cost. Moreover, they receive small nonfood gifts to reinforce trial retention.

The dietary intervention started 4 to 6 weeks after the baseline phenotyping at visit (V) 0 (V0). All participants are asked to stabilize their individual body weight if possible. Further details of nutritional sessions as well as the dietary protocol of the intervention and control group are described in the Supplemental Digital Content (for used NutriAct foods in the intervention group see Supplemental Digital Content (Table S1)).

In addition, participants of both groups are regularly informed regarding their health status as well as crucial abnormal results of the phenotyping during the trial via telephone calls and written reports to further reinforce trial retention. Additional telephone assessments at month 18 and 30 are performed by the study staff to reflect the individual health status and to answer further questions of the participants.

### 3.5 Outcomes

In order to investigate the effect of the above-described nutritional strategy for healthy aging in middle-aged and elderly people, the primary outcome measure is defined as a composite endpoint of age-related disorders including cardiovascular morbidity and age-related impairment of cognitive function as well as lean body mass and muscular function.

In detail, this includes:

- Cardiovascular morbidity
  ∘ Newly developed heart failure as defined by development of left ventricular (LV) ejection fraction <50% or hospitalization for heart failure
  ∘ Incidence of CHD, myocardial infarction, cardiovascular revascularization, stroke
  ∘ Increase of blood pressure by 10 mm Hg systolic or 10 mm Hg diastolic or more
- Decrease of lean body mass (as a known estimate of muscle mass assessed by Dual-energy X-ray Absorbtiometry (DEXA)) by 3 kg or more
- Muscular function
  ∘ Decrease of SPPB sum score by 1 point or more
  ∘ Decrease in hand grip strength by 3 kg or more
- Cognitive function (impairment of at least one of the cognitive domains memory, processing speed or executive function)
  ∘ Memory: Decrease in forward digit span or backward digit span of one or more digits
  ∘ Processing speed: Increase in Stroop test time of 25 seconds or more
  ∘ Executive function: Increase in Trail Making Test A time of 15 seconds or more.

The study should provide evidence that the intervention supports healthy aging by decreasing the rate of the composite endpoint, implying that it mitigates at least one of the sub-endpoints over the 3 studied years.

The secondary outcome measures are: the distribution of intraabdominal, intrahepatic and intramuscular fat, change of insulin sensitivity, impairment of cardiac function, CHD, revascularization, myocardial infarction, stroke, increase of blood pressure or antihypertensive treatment, decline of muscle strength, lean body mass, frailty, physical activity, cognitive function, estimates of (health related) quality of life (QoL), inflammatory markers, cardiovascular and metabolic risk markers, biomarker responses to diet-induced changes, energy expenditure, depression, mRNA and protein expression in subcutaneous adipose tissue, gut microbiome, effects on individual dietary pattern as well as adherence to the diet. These analyses will also include gender specific aspects.

### 3.6 Recruitment strategy

Potential participants were recruited through advertisement in public media (intranet, newspaper) and brochures. The first participant was enrolled in June 2016 and recruitment was completed in July 2018.

### 3.7 Participant timeline

Phenotyping of all participants was performed at baseline (V0). Further phenotyping was performed 3 (V1), 6 (V2), 12 (V3), 24 (V4) and 36 (V5) months after start of dietary intervention. Figure 1 illustrates the study flowchart with time points of phenotyping and sessions of nutrition counselling within the trial for intervention and control group (for detailed time points of nutrition sessions see Supplemental Digital Content).

## 4 Methods: assignment of interventions

### 4.1 Allocation

After initial characterization, participants were randomized with a 1:1 allocation ratio into an intervention group or a control group. Randomization was performed using stratified randomization. The stratification criteria were: gender; known CVD (CHD, PAD, myocardial infarction, stroke); heart failure (NYHA ≥2 or NT-proBNP >300 ng/l if no atrial fibrillation is present); arterial hypertension, known type 2 diabetes or newly diagnosed type 2 diabetes (based on results of oral glucose tolerance test at V0); cognitive impairment (MoCA test <26); SPPB score <10 (all yes or no). In case of inclusion of 2 subjects of the same family, the first family member was randomized according to the protocol and the second family member was allocated to the same group. An adaptive randomization was performed to balance for the above-mentioned stratification criteria. Allocation concealment was ensured, as the service did not release the randomisation code until the subject had been recruited into the trial, which took place after all baseline measurements had been completed. Due to the nature of the intervention, participants could not be blinded regarding group assignment.

## 5 Methods: data collection, management, and analysis

### 5.1 Data collection methods

All data are collected by means of electronic case report form (eCRF) using REDCap. All measurements are performed in accordance with standard operating procedures (SOPs) that were developed or adapted for this study and are conducted by trained and certified study staff. All intended phenotyping procedures and SOPs were harmonized between study centers. For quality assurance and standardized assessment of data, standardized study document sets are provided, including (web-based) data collection instruments, examination scheduling forms and standard sets with biosample storage material. All study nurses were trained by qualified staff in all phenotyping procedures performed within the trial. Data collection comprises medical history (including adverse events), drug history, alcohol consumption and smoking behaviour, family medical history, socio-demographic status and physical activity. All subjects undergo a comprehensive physical examination and data collection regarding cardiovascular, metabolic, muscular and cognitive function. All outcome measures for the assessment time points are presented in Table 1.

**Table 1:**
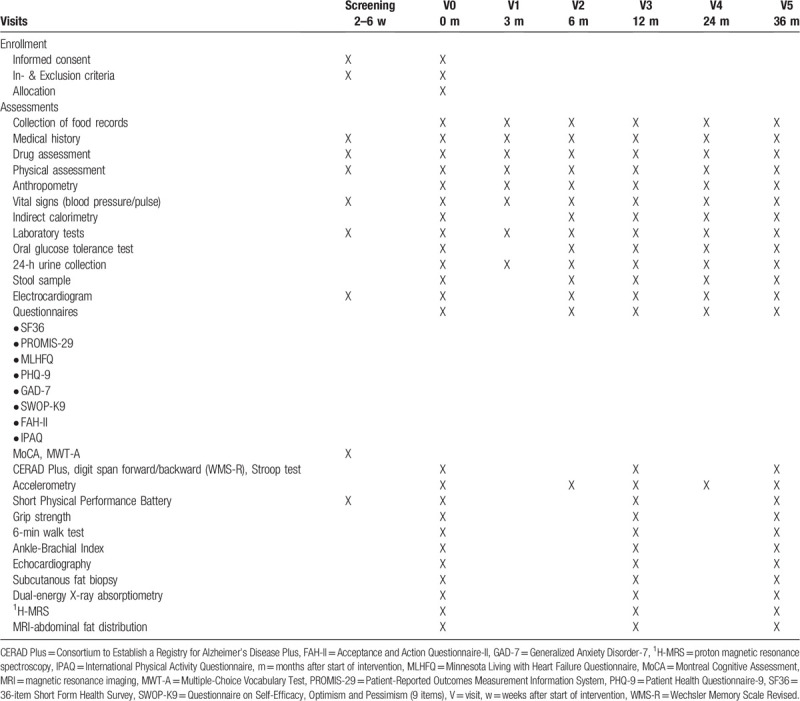
NutriAct schedule of enrollment and assessments.

#### 5.1.1 Anthropometry

Following a 10-hour overnight fast, all subjects are examined at 8:00 am. Body weight (to the nearest of 0.1 kg) and height are measured with a digital column scale with integrated stadiometer (Charité: Seca, Hamburg, Germany; DIfE: Soehnle, Nassau, Germany). The body mass index (BMI) is calculated (the weight in kilograms divided by the square of the height in meters). Waist and hip circumference are measured 2 times and the means are calculated.

#### 5.1.2 Cardiovascular assessment

Blood pressure is measured 3 times at the left arm and one time at the right arm with the participants sitting for at least 5 minutes and always using the same sphygmomanometer. In detail, a digital sphygmomanometer is used at the study center of the Charité (Carat professional, boso, Jungingen, Germany) and a manual sphygmomanometer at the study center of DIfE (boso, Jungingen, Germany). Means of measured values are calculated. A 12-channel-electrocardiogram is performed in all participants at each visit. Vascular status is being assessed by ankle-brachial index^[37]^ (ABI-system 100, boso, Jungingen, Germany). Here, one measurement is taken after a resting period of 5 minutes in lying position. Moreover, all subjects are examined at rest using an EPIQ 7 (Philips Healthcare, Germany) ultrasound machine for thransthoracic echocardiographic measurements. Chamber dimensions are evaluated using standard procedures, including LV mass index and left atrial volume index. The assessment of diastolic function includes pulsed-wave doppler measurements, tissue doppler measurements in 4-chamber view over at least 3 cardiac cycles and measurement of tricuspid regurgitant jet as recommended by the American society of echocardiography.^[38,39]^ Volume changes of the left ventricle are obtained by a 3-dimensional acquisition using full-volume assessment over 4 cardiac cycles. A Philips QLab software is used for post-acquisition volume analysis and measurement of LV end-diastolic volume, end-systolic volume, stroke volume, cardiac output and ejection fraction. The analysis of LV strain using 2D speckle-tracing echocardiography is determined as the average value of the longitudinal negative strain peak during LV contraction from all segments of the left ventricle in the apical 4-chamber, 3-chamber and 2-chamber views. LV strain measurements are performed at frame rates of 50 to 80 frames/s, averaging 3 measurements, and using the onset of the QRS as the referent point. The analysis is performed by 2 independent investigators who are blinded for all information and validation is proved in the Echocardiography Core Lab of the Department of Cardiology at the Charite Berlin, Campus Virchow Klinikum.

#### 5.1.3 Metabolic phenotyping

Resting energy expenditure (REE) and whole body substrate utilization are being measured for 30 minutes by indirect calorimetry using a ventilatory hood (Charité: Quark RMR, Cosmed, Sundern, Germany; DIfE: Vmax Encore Metabolic Cart, CareFusion, Yorba Linda, California, USA) after a 20 minute resting period.

Moreover, subjects undergo a fasting venous blood sample collection followed by an oral 75 g glucose tolerance test. Further blood samples are taken after 15, 30, 60, 90, 120 and 180 min.

Abdominal subcutaneous tissue biopsies (approximately 5.0–10.0 g) are obtained in a subgroup in fasting state at baseline, after 1 and 3 years by needle biopsies from the periumbilical region using an aspiration through a 12 G needle with a side cutout and round end. After anesthetization of skin with 1% lidocaine without epinephrine, a skin incision (3–4 mm) is made and adipose tissue biopsies are obtained. Samples are washed twice in 0.9% NaCl to separate adipose tissue from blood and immediately snap-frozen in liquid nitrogen and stored at −80 °C until further laboratory analysis.

#### 5.1.4 Self-administered questionnaires

Health related QoL is assessed by 36-item Short Form Health Survey (SF-36)^[40]^ and Patient-Reported Outcomes Measurement Information System (PROMIS-29) v2.0 Profile (www.healthmeasures.net).^[41,42]^ Disease-specific QoL is assessed by Minnesota Living with Heart Failure Questionnaire (MLHFQ).^[43]^ The questionnaires Patient Health Questionnaire-9 (PHQ-9),^[44]^ Generalized Anxiety Disorder-7 (GAD-7),^[45]^ Self-Efficacy, Optimism and Pessimism (9 items; SWOP-K9)^[46]^ and Acceptance and Action Questionnaire-II (FAH-II)^[47]^ are used for evaluating depression or psychosomatic state, respectively. Physical activity is assessed by International Physical Activity Questionnaire (IPAQ; www.ipaq.ki.se).^[48]^ All questionnaires are usually completed electronically on mobile devices. A paper version or support for completing the questionnaires is provided if necessary.

#### 5.1.5 Cognitive assessment

To characterize the cognitive status the German version of MoCA^[49]^ and Multiple-Choice Vocabulary Test (MWT-A)^[50]^ was performed for screening. Cognitive function is specifically assessed by a standardized neuropsychological test battery. This test battery comprises subtests of the extended version of the Consortium to Establish a Registry for Alzheimer's Disease (CERAD) test battery^[51]^ (CERAD-Plus test battery, Revised edition 2005, Memory Clinic Basel, Switzerland, www.memoryclinic.ch) as well as digit span forward and backward of the Wechsler Memory Scale (WMS) Revised^[52]^ and a Stroop color-word-interference test.^[53]^

#### 5.1.6 Evaluation of physical function

We perform SPPB to analyze physical function (including muscle strength) of the lower extremity, which consists of a balance test, gait speed test and 5-chair rise test.^[54]^ Grip strength is measured to assess muscle strength of the upper extremity using Jamar Plus Digital Hand Dynamometer (Sammons Preston, Bolingbrook, Illinois, USA). Further details for evaluation of motor function are described in the Supplemental Digital Content. The 6-minute walk test^[55]^ is being conducted to assess exercise capacity (in a subgroup of 252 participants). Frailty and physical activity are evaluated with The Frailty Phenotype Criteria according to Fried^[56]^ and with accelerometry (wGT3X-BT, ActiGraph, Pensacola, Florida, USA), respectively. Subjects are instructed to wear the accelerometer for 7 days on the right waist above or underneath clothing and to pursue daily activities as usual.

#### 5.1.7 Imaging

Body composition, including lean body mass, and bone density are measured using DEXA (Charité: QDR Discovery W, DIfE: QDR Explorer W; Hologic Canada ULC, Mississauga, Canada).

Magnetic resonance examinations are performed in every participant on a 1.5-T whole-body scanner (Magnetom Avanto, Siemens Healthcare, Erlangen, Germany) for quantification of abdominal fat depots by axial magnetic resonance imaging (MRI) as well as quantification of intrahepatic lipids (IHL) and intramyocellular lipids (IMCL; in a subgroup) in tibialis anterior and soleus muscle by localized proton magnetic resonance spectroscopy (^1^H-MRS). Analysis of MRI and ^1^H-MRS data is carried out in cooperation with the Institute for Diabetes Research and Metabolic Diseases (IDM) of the Helmholtz Center Munich at the University of Tübingen. For quantification of abdominal fat depots, an axial T1-weighted fast spin echo technique is applied as described by Machann et al.^[57]^ Visceral adipose tissue is quantified from femoral head to thoracic diaphragm and nonvisceral abdominal adipose tissue integrating subcutaneous, intermuscular, and intrathoracic fat from femur to humerus - both in liters - by an automated segmentation algorithm based on fuzzy clustering.^[58]^ Additionally, adipose tissue on the level of femoral head and humerus are determined, which have shown to be representative for adipose tissue of the lower and upper extremities, respectively.^[59]^ IHLs are quantified by a single voxel stimulated echo acquisition mode (STEAM) technique with a voxel (volume of interest, VOI) size of 30 × 30 × 20 mm^3^ in the posterior part of segment 7^[60]^ and IHLs are given as ratio of fat (methylene + methyl resonances at 1.3 and 0.9 ppm, respectively) divided by the sum of water (at 4.7 ppm) and fat resonances, respectively. IMCL are determined from a VOI (11 × 11 × 20 mm^3^) placed in the most extended part of the right calf by a STEAM technique without and with water suppression. Regions with macroscopically visible fatty septa were carefully excluded from the VOI wherever possible. IMCL are quantified in arbitrary units by calculation of the methylene signal integral of IMCL at 1.3 ppm in the water suppressed spectrum in relation to the water signal at 4.7 ppm.^[61]^

#### 5.1.8 Collection of nutritional data

All participants complete open food records on 3 consecutive days, including one weekend day, 10-14 days before each visit (see Supplemental Digital Content (Fig. S1)). Therefore, participants are asked to possibly weigh or alternatively use a standard household measure (e.g. a tablespoon) as an estimate of consumed foods and beverages. These data are converted to intake of energy and nutrients through nutrient calculation software (Prodi 6.5 Expert; Nutri-Science GmbH, Freiburg, Germany). To strengthen adherence to the intended dietary pattern and to test different methods for dietary assessment, participants of both groups are additionally asked to fill out a modified web-based 24-hour food list^[62]^ on 21 random days during the whole study period (further details in the Supplemental Digital Content). However, only the open 3-day food records collected at each visit are used for dietary recommendations by the nutritionists. To monitor the intended stabilization of body weight, participants are also asked to record their body weight on the open food records.

#### 5.1.9 Laboratory assessment

Capillary blood glucose is immediately measured by point-of-care method using the glucose oxidase method (Charité: Dr. Müller Super GL 2, Freital, Germany; DIfE: Biosen C-line, EKF-diagnostic GmbH, Barleben, Germany). Glucose is additionally measured in fluoride plasma (GlucoEXACT tubes; Sarstedt, Nuembrecht, Germany) performed on Cobas Mira (Roche Diagnostics, Mannheim, Germany). Preprocessing and intermediate storage of blood samples are being performed at both study centers. Blood samples are being centrifuged and frozen immediately at −80 °C. Blood count and plasma NT-proBNP are measured immediately performed on Sysmex XN-9000 (Sysmex, Norderstedt, Germany) and cobas e602 module (Roche Diagnostics, Mannheim, Germany) using Roche Elecsys Immunoassay, respectively. Protein, transaminases, potassium, sodium, calcium, creatinine, urea, uric acid, triglycerides, cholesterol and high-density lipoprotein (HDL)-cholesterol are measured in serum using standard laboratory methods performed on ABX pentra 400 (HORIBA ABX SAS, Montpellier, France). LDL-cholesterol is calculated using the Friedewald formula. Glycated hemoglobin (HbA1c) is quantified in EDTA blood using spectrophotometry performed on ABX pentra 400 (HORIBA ABX SAS, Montpellier, France). Serum insulin and c-peptide are measured using enzyme-linked immunosorbent assays (ELISA; Mercodia, Uppsala, Sweden) (intra-assay CV 2.8%–4.0% (insulin) and <4.8% (c-peptide), inter-assay CV 4%–5% (insulin) and <6.8% (c-peptide)). Non-esterified fatty acids (NEFAs) are quantified in serum using a commercially available colorimetric assay (NEFA HR2, Wako, Neuss, Germany) (intra-assay CV <1.5%, inter-assay CV <5%). CRP is measured in serum using ABX Pentra CRP CP (Horiba ABX SAS, Montpellier, France) (intra-assay CV 0.74%–4.1%, inter-assay CV 2.17%–4.31%). Buffy coat samples are obtained from EDTA blood for further analysis. Isolation of peripheral blood mononuclear cells (PBMC) for further analysis is performed in a subsample using BD Vacutainer CPT tubes (BD Bectin Dickinson GmbH, Heidelberg, Germany). Isolation of RNA is performed using PAXgene blood RNA tubes (BD Bectin Dickinson GmbH, Heidelberg, Germany). First, these tubes are kept at room temperature for 2 hours. Then, after intermediate storage at −20 °C for at least 24 hours, they are stored at −80 °C for further analysis.

Stool samples are collected by the participants at home in faeces containers (Sarstedt, Nuembrecht, Germany), stored at participants’ home at −20 °C and at the study center at −80 °C until shipment to the laboratory for further analysis. 24-hour urine samples are obtained in containers (Sarstedt, Nuembrecht, Germany) and stored at −80 °C.

#### 5.1.10 Data management

All study relevant data are stored pseudonymized in digital form (eCRF). Electronic data is managed on protected servers with access restrictions. Participants’ files are stored in numerical order and at a secure place, only accessible to authorized persons (Principal Investigator, study physicians, study nurses), for a period of 15 years after completion of the study. Quality control is being ensured through regular data checks. For example, plausibility controls of the online data are being performed by frequency distribution checks of outcome measures on a regular base.

### 5.2 Sample size

The sample size was planned based on previously reported data of a large RCT comparing different counselling strategies over 30 months.^[63]^ Sample size calculation for a log rank test was conducted in accordance with the primary endpoint. The sample size calculation was performed with nQuery Advisor V7.0. From the literature,^[64–71]^ the cut-off values for the primary endpoint were selected such that in each component, an incidence of 10% to 15% is expected over 3 years. Depending on the correlation of the events, this may lead to a proportion of event-free patients of about 27% to 80% at the end of the observation period. We assume an improvement of around 10% to 13%, and therefore a reduction in events by the interventions. This difference will be considered realistic and clinically relevant. This corresponds to a hazard ratio of 1.42 to 2.11 which, at 80% power and a 2-sided alpha of 5%, results in sample sizes of 200 to 226 patients per group. Calculating a drop out of approximately 10% to 15%, 500 subjects should be at least included.

### 5.3 Statistical analysis

The primary outcome measure is defined as a composite endpoint including cardiovascular morbidity and age-related impairment of cognitive function as well as lean body mass and muscular function as described above. We aim to show that this intervention will decrease the rate of the composite endpoint, implying that it mitigates at least one of the sub-endpoints during the 3 studied years. The main analysis will be conducted with a log rank test for superiority of the intervention. A *P* value <.05 is considered statistically significant. Possible effect modifiers and confounders will be included in the cox regression analyses as needed to explain group differences. Kaplan-Meier curves are used to present the effects graphically. The analysis will be carried out in the intention-to-treat population. Missing values will be replaced by multiple imputation wherever necessary. Moreover, a per-protocol analyses will also be performed. Secondary endpoints will be analysed descriptively according to their presence and distribution using the usual statistical parameters. Both parametric and non-parametric tests will be used depending on the distribution of data. All *P* values from the secondary analyses will be considered exploratory and non-confirmatory.

## 6 Methods: monitoring

### 6.1 Data monitoring

A data monitoring committee is not established during the trial since this clinical trial does not include any prescription of new medication and usual care as well as previously prescribed medication is not affected during study participation. Therefore, the trial has a minimal risk for induction of serious adverse events.

### 6.2 Adverse events monitoring and reporting

A detailed monitoring plan is included as part of the protocol in each visit. These plans include documentation of any adverse event (AE) or serious adverse event (SAE) on case-report forms (eCRF) whether it is an unfavorable and unintended sign (including an abnormal laboratory finding, for example), symptom, or disease, whether or not it is considered related to the intervention. AEs and SAEs are documented throughout the complete follow-up period. SAEs with potential causal relationship with the intervention are reported to the Principal Investigators within 24 hours. In addition, participants are withdrawn from the study if it is medically indicated in the opinion of the Principal Investigator.

## 7 Discussion

Age-related health decline such as the development of cardiovascular diseases, sarcopenia and cognitive decline is a growing challenge as the mean age of our population increases. Up to date, the optimal macronutrient composition to counteract age-related changes remains unclear, especially as large RCTs in non-mediterranean populations are still lacking and some data is controversial, for example, concerning the beneficial effect of PUFA on cardiovascular risk factors^[21,24,28]^ or the recommendable amount of protein.^[12]^ Predominantly observational or non-randomized intervention studies indicate benefit of high protein (especially plant protein) intake and replacement of MUFA and PUFA instead of SFA on improvement of cardiovascular, muscular and cognitive endpoints in elderly.^[8,9,16,18]^ Together with a high fibre content and a low glycemic index, such a pattern may also lead to a lesser deterioration of age-related metabolic status, for example, a reduced risk for development of type 2 diabetes.^[30,33]^ Given these data indicating a beneficial effect of specific nutritional components, we decided to analyze the effect of a complex dietary pattern including high intake of MUFA and PUFA, plant proteins, improved fibre content as well as a low glycemic index (the NutriAct pattern) within a long-term trial. To our best knowledge, this is the first long-term randomized controlled intervention study with large sample size in a middle-aged and elderly German population analyzing the effects of such a dietary pattern. Long-term modification of nutritional behavior is a well-known challenge. Therefore, the supplementation of specific designed food products in conjunction with regular nutritional sessions to support its implementation, acceptance and thus long-term adherence to this dietary pattern is a key component of the NutriAct approach. Given the high acceptance of rapeseed oil in the local population as well as the promising effects of short-term consumption of rapeseed oil on cardiovascular and metabolic risk markers,^[72]^ we focus on supplementation of rapeseed oil as a preferred source of dietary fat.

The NutriAct approach will be compared to usual care based on local standard care which also includes dietary recommendations of the DGE.^[34,36]^ As the NutriAct dietary pattern is substantially different from current local food consumption, a larger number of nutrition sessions is included compared to the control group.

The main objectives of this study are to analyze the long-term effect of NutriAct dietary pattern on healthy aging, particularly cardiovascular morbidity, cognition and sarcopenia. Further objectives are to investigate gender specific effects as well as effects on other age-related phenotypes such as bone density, quality of life or glucose metabolism. Therefore, a complex state of the art phenotyping is implemented in this study. These phenotyping procedures, which also include adipose tissue biopsies, DEXA and MRI/H-MRS scan, will allow a complex analysis of tissue specific metabolic responses. Associations between measured biomarkers at baseline as well as biomarker changes during the dietary intervention and concomitant alteration of metabolic, cognitive and cardiovascular status will further help to identify predictors of age-related health and potential mechanisms involved in those changes.

The findings of this RCT will contribute to define the optimal macronutrient composition in the context of healthy aging and to the implementation of a realizable healthy dietary pattern in a German population.

## Acknowledgments

We thank S. Jürgens, N. Huckauf, C. Kalischke, A. Borchert, K. Ritter, S. Ernst, K. Warnke, P. Großmann, T. Mikhailova, T. Brechlin and U. Redel for excellent technical assistance as well as F. Schwerin, R. Lifka, N. Stobäus, L. Napieralski, M. Hannemann, E. Wehrstedt, S. Schröter and D. Zschau for the excellent support regarding phenotyping. Furthermore, we thank E. Siebenhühner, S. Schönfuss and C. Heerling for conducting nutrition counselling and U. Harttig, E. Kohlsdorf and K. Treu for support regarding the modified web-based 24h-food list. We also thank E. Kohlsdorf, M. Osterhoff, H. Piechot and A. Abel for contributing substantial support in data management as well as N. Külzow and N. Stobäus for support concerning psychologic, cognitive and metabolic phenotyping. Our special thanks also go to the departments of radiology, Charité Campus Virchow-Klinikum and Ernst von Bergmann Klinikum, Potsdam. Moreover, we thank D. Baier, S. Sevenich and U. Rzeha (NutriAct innovation office) for managing contacts and negotiations with the SMEs. We thank the following SMEs for development and delivery of specific food supplements in the intervention group: rapeseed oil (Brökelmann & Co - Oelmühle GmbH &Co, Hamm), oil cake and base mix for muesli (Kanow-Mühle Sagritz, Golßen), bread rolls (DewiBack Handels GmbH, Berlin; J. Rettenmaier & Söhne GmBH + CoKG, Rosenberg), protein enriched pasta and flakes (IGV GmbH, Nuthetal). We thank Dieckmann GmbH + CoKG, Rinteln, and Zweiglein UG, Potsdam, for delivery of barley flakes and muesli, respectively, for use in the control group. Food supplements are also designed in cooperation with IGV GmbH and institute of food technology at TU Berlin.

## Author contributions

KM, AFHP, SH and JS designed the study. KM, AFHP, TG, MB and JS designed and organized the study logistics. CW, KA, SH, AE, UP, SS, KH, JM and CG researched data. CW, KM, JS, UP, JM, KH, CG and AP wrote the manuscript. All authors critically read and edited several drafts before submission. All authors read and approved the submitted version. AFHP, CG, JS and KM designed the dietary intervention.

## Supplementary Material

Supplemental Digital Content

## Supplementary Material

Supplemental Digital Content

## Supplementary Material

Supplemental Digital Content
