# Supplementary material for: Long-term effects of a food pattern on cardiovascular risk factors and age-related changes of muscular and cognitive function
Source: Medicine (Baltimore). 2020 Sep 25;99(39):e22381. doi: 10.1097/MD.0000000000022381 (PMC7523819; doi:10.1097/MD.0000000000022381)
Supplement: Supplemental Digital Content [file medi-99-e22381-s002.docx]

**Table S1.** Food products used in the intervention group.

|  | **Protein flakes** | **Protein pasta** | **Rolls with a topping of sunflower seeds** | **Rolls with a topping of pumpkin seeds** | **Flaxseed oilcake** | **Pumpkin seeds oilcake** | **Rape-**  **seed oil** | **Base mix muesli** |
| --- | --- | --- | --- | --- | --- | --- | --- | --- |
| **energy**  **[kJ/kcal]^1^** | 1700/  402 | 1449/  343 | 1108/  265 | 1137/  273 | 1528/  369 | 1869/  445 | 3404/  828 | 2384/  569 |
| **protein [g]^1^** | 59.5 | 23.9 | 17.5 | 17.9 | 32.0 | 56.0 | 0 | 28 |
| **carbohydrates [g]^1^**  of which sugar [g] | 25.2  9.4 | 52.9  3.6 | 24.8  0.6 | 22.4  0.4 | 3.0  3.0 | 12.0  2.0 | 0  0 | 10  1 |
| **fat [g]^1^**  of which SFA [g]  of which PUFA [g]  of which MUFA [g] | 6.4  1.4  3.1  1.9 | 1.9  0.3  0.7  0.7 | 8.9  0.8  n/a  n/a | 10.5  0.3  n/a  n/a | 19.0  2.0  13.0  n/a | 19.0  4.0  9.0  n/a | 92.0  6.5  25.5  60 | 47  6  18  10 |
| **dietary fibre [g]^1^** | 2.9 | 9.1 | 3.9 | 4.1 | 32.0 | 19.0 | 0 | 23 |
| **salt [g]^1^** | 1.6 | 0.6 | 1.1 | 1.1 | 0 | 0 | 0 | <1 |

*kJ*, kilojoule; *kcal*, kilocalories*; g*, gram; ^1^per 100g; SFA, saturated fatty acids; PUFA, polyunsaturated fatty acids; MUFA, monounsaturated fatty acids; n/a, not available
