# Supplementary material for: Long-term effects of a food pattern on cardiovascular risk factors and age-related changes of muscular and cognitive function
Source: Medicine (Baltimore). 2020 Sep 25;99(39):e22381. doi: 10.1097/MD.0000000000022381 (PMC7523819; doi:10.1097/MD.0000000000022381)
Supplement: Supplemental Digital Content [file medi-99-e22381-s003.doc]

**Supplemental Digital Content**

**Assessment of physical function**

Physical function of the lower extremity is assessed by Short Physical Performance Battery (SPPB) measuring three domains of physical function: maintenance of standing balance, usual gait speed and lower extremity strength^1^. The individual component contributes up to 4 points in the overall score, so the SPPB scores range from 0 to 12, with higher scores reflecting better lower extremity physical function. The balance test includes side-to-side, semi-tandem and tandem stand test. No aid is used and participants are barefoot on even ground. The instructor demonstrates foot positions before without practice by the participants. Participants keep eyes open during the tests. For side-to-side test, participants stand with feet together side-by-side for 10 seconds. It is followed by the semi-tandem with heel of one foot against side of big toe of the other for 10 seconds. Finally, the tandem stand is performed with feet aligned heel to toe for 10 seconds. The test is cancelled when participants move from the intended position, need aid or are about to fall. Locomotion is tested by gait speed test. Therefore, the required time to manage a given walking distance of four meters is measured in seconds in usual walking speed. Walking aids are used as necessary in daily life. The best run of two performances is being evaluated. Lower extremity strength is tested by five-chair rise test. The participant stands up as quickly as possible five times from a chair of usual height (approximately 46 cm seat height) and without armrests. Arms should be crossed in front of the chest. Time is measured in seconds including milliseconds, starting with the first standing up and ending when the participant has reached standing position for the fifth time. The participant is repeatedly and empathically encouraged to perform the task as quickly as possible before and during the test. First, one test run is performed to check if the participant can safely manage to stand up without using arms. Only then, the real test starts. It is stopped if participants use their arms, become respiratory insufficient or take longer than one minute.

In addition to SPPB hand-grip strength is measured using a Jamar Plus Digital Hand Dynamometer (Sammons Preston, Bolingbrook, Illinois, USA) to assess upper extremity strength. The participant sits on a chair with the arms unsupported and the shoulder joint abducted and in neutral position. The elbow joint is 90° flexed, the forearm is in neutral position and the wrist is in 0-30° dorsal extension and in 0-15° ulnar deviation. In this position, a test round is conducted. The participant should compress the digital dynamometer as firmly as possible with the dominant hand, this sequence is repeated with the non-dominant hand. Then, measurements are taken three times with the dominant hand and means are calculated. One measurement is taken with the non-dominant hand. Measures of grip strength can range from 0 to 90 kg with age- and sex-specific reference values.

**Dietary intervention protocol**

**Generalities**

Two different dietary patterns are compared in this randomized controlled trial. The dietary intervention will be performed for 36 months and is conducted by professional dieticians. A detailed session schedule and assessment of food intake are depicted in figure S1. Nutrition counselling is performed in closed groups including 6 - 10 participants and lasts approximately two hours. The intervention strategy is mainly food-based. Therefore, the individual topics of the counselling sessions were designed to support the participants to change their eating habits in line with the recommended food pattern. Practice-oriented information about foods and various strategies designed to change eating habits are mainly discussed in the groups. This includes enhancement of self-responsibility for one`s own health and strengthening of the self-efficacy of the participants. Priority is given to problems, questions and comments of the participants. In the beginning, each participant received a folder with detailed information about the dietary recommendations according to group assignment (e.g. information about the food pattern, cooking recipes with nutritional values for breakfast, lunch and dinner). Participants complete open three-days food records before each visit (V) (see figure S1) and receive individual food-based and nutrient-based analyses of these food records. The food-based analysis includes the allocation of the consumed foods to special food groups, e.g. vegetables, dairy products. The nutrient-based analysis comprises the distribution of macronutrients (carbohydrates, fat, protein), the intake of the various fatty acids (saturated fatty acids (SFA), monounsaturated fatty acids (MUFA), polyunsaturated fatty acids (PUFA)), vitamins (A, E, K, B1, B2, B6, B12, C, pantothenic acid, niacin, folic acid, biotin), minerals (calcium, zinc, iron, potassium, magnesium, sodium), dietary fibre, cholesterol, water and alcohol. All participants agreed to discuss the results of individual food records and individual nutrition-related blood values (blood values are only discussed in the DIfE study center) in the group sessions.

To strengthen adherence to the intended food pattern and to test different methods for dietary assessment, participants of both groups are additionally asked to fill out a modified web-based 24-h food list^2^ on 21 random days during the whole study period. The original food list was modified by adding questions on numbers of portions and the day time when the item was eaten (during the morning, before noon, noon, after noon, evening, night), and by adding further food items relevant for the intervention (e.g. protein enriched bread rolls, -noodles).

**Description of dietary pattern in intervention and control group**

**NutriAct Intervention group**

The dietary pattern of the intervention group consists of 35-40%E total fat (SFA ≤ 10 %E, MUFA 15-20 %E and PUFA 10-15 %E), 15-25 %E protein (emphasis on plant proteins), 35-45 %E carbohydrates and at least 30 g dietary fibre per day. The pattern is defined by the composition of the macronutrients. In order to achieve the recommended pattern several foods (“NutriAct foods”) were designed in accordance to the intended dietary pattern and are provided to the participants free of charge (table S1). Participants are also advised to buy other foods high in protein, dietary fibre or PUFA. In general, participants of the intervention group are advised to consume foods according to the following main recommendations: 20 g (for women) and 30 g (for men) rapeseed oil, 10 g linseed- or pumpkin seed oil cake, 30 g nuts or seeds (preferably walnuts), 400 g vegetables and salad, 250 g fruits, 200 – 250 g milk or dairy products and 50-60 g cheese per day. Furthermore, a weekly intake of 40 g protein flakes, 4 protein rolls or 4 x 30g of base mix for muesli, 1 portion of protein noodles, 2 portions of legumes, 2 portions of fish and up to 300 – 600 g low fat meat and sausages is advised within this group. 21 group sessions are conducted so that participants become accustomed to the recommended dietary pattern.

**Control group (usual-care group)**

A balanced health-promoting diet is advised in the control group in accordance to the recommendations of the German Society of Nutrition (DGE)^3,4^. The main aim is the reduction of a high intake of total fat and SFA and only a moderate sugar intake. The distribution of the macronutrients is: 55 %E carbohydrates, total fat 30 %E (SFA ≤ 10 %E, MUFA ≥ 10 %E, PUFA 7-10 %E), 15 %E protein. The food-based guide values per day are: 4-6 portions of cereal products (200 – 250 g), preferably whole grains; 1 portion of potatoes, rice or noodles; 3 portions of vegetables and salad (400 g) and 2 portions of fruit (250 g); 200 – 250 g milk or dairy products and 50-60 g cheese, preferably low fat varieties; 10-15 g oil and 15-30 g spreadable fat; approximately 1,5 l calorie-free or low-calorie beverages. Additionally, the food-based guide values per week are: 300 – 600 g low fat meat and sausages; 2 portions of fish; up to 3 eggs. Five group sessions are conducted in the control group. Each participant receives one packet of high carbohydrate muesli (435 g) and two packets of barley flakes (250 g) and barley as an alternative to rice (250 g), respectively, throughout the entire study period. Moreover, subjects in the control group receive small non-food gifts.

**Overview of nutrition sessions for intervention group**

**1. Nutrition session (start of intervention, 4-6 weeks after V0)**

The aims and the concept of the study are again presented to the participants. The NutriAct food pattern is explained in detail. Subjects are informed about different kinds of fatty acids (SFA, MUFA and PUFA) and their differential effects (e.g. adverse effects of SFA) on blood cholesterol levels. In this context, participants are trained to recognize the amount of SFA on food labels. Over the next three weeks, participants are encouraged to limit their intake of SFA to 19 g (women) or 23 g (men) per day and to complete food records over 20 days to estimate and reflect the intake of SFA. Moreover, they are instructed to completely avoid sweets and alcoholic or sweet beverages over the next three weeks in order to maintain a balanced overall energy intake while increasing the intake of plant-based foods with high content of healthy fats.

**2. Nutrition session (after one week of intervention)**

The beneficial impact of a diet high in unsaturated fatty acids, e.g. on blood cholesterol levels, is explained in this session. Participants learn options to increase the proportion of PUFA in their diet. In this context, the higher energy content of fat in comparison to carbohydrates and proteins is outlined.

**3. Nutrition session (after three weeks of intervention)**

Proteins and protein function such as building and maintaining muscle mass, synthesis of hormones and promoting satiety are explained in this session. Furthermore, protein content of different food products, also in relation to fat content, as well as possibilities for combination of different plant-based and animal products, or exclusively plant based products, are presented.

**4. Nutrition session (after six weeks of intervention)**

In this unit, carbohydrate uptake and its effects on glucose metabolism are roughly explained. Thereby, the beneficial impact of a high dietary fibre content in carbohydrate-containing foods is emphasized.

**5. Nutrition session (after 10 weeks of intervention)**

Different sorts of carbohydrates with particular focus on sugar and other sweeteners are presented in this session. In this context, nutritional labeling of packaged food, especially the declaration of "carbohydrates" and "of which sugar", is explained with examples.

**6. Nutrition session (after 15 weeks of intervention)**

In this session, individual V1 food records and nutrition-related blood values are discussed in detail. Moreover, practice-oriented information to achieve the recommended food pattern is collected in the group.

**7. Nutrition session (after 19 weeks of intervention)**

Different sugar-related terms such as "reduced sugar content", "low in sugar" and „no added sugar" are explained in this session. In this context, subjects learn more about sugar substitutes that are low in energy and a moderate intake of sugar and its substitutes is encouraged.

**8. Nutrition session (after 23 weeks of intervention)**

In this session, dietary fibre, its sources (with concrete examples of foods particularly rich in fibre) and its effects on metabolism are presented.

**9. Nutrition session (after 27 weeks of intervention)**

In this session, individual V2 food records and nutrition-related blood values are discussed in detail. Moreover, practice-oriented information to achieve the recommended food pattern is collected in the group.

**10. Nutrition session (after 8 months of intervention)**

Different kinds of vitamins as essential nutritional ingredients for maintaining physical functions and health are presented in this session. Here, the distinction between water- und fat-soluble vitamins is outlined with reference to regulation and storage in the human body. Subjects learn possibilities to combine dietary sources fat-soluble vitamins with appropriate food products in order to facilitate their uptake, such as carrots as a source of vitamin A with different kinds of oil.

**11. Nutrition session (after 10 months of intervention)**

Minerals as essentials nutrients and possibilities to cover these needs through a varied and balanced diet are presented in this session. Here, especially minerals often consumed in insufficient or excessive quantities such as calcium or sodium are in particular focus by providing concrete recommendations, e.g. consumption of three low-fat milk products to support an adequate calcium intake.

**12. Nutrition session (after 12 months of intervention)**

The body’s water balance and risks of dehydration, such as impaired renal function or blood pressure dysregulation, are explained in this session. Thereby, participants are informed that thirst may decrease with age and that a fluid intake of 1.5 - 2 liters a day, avoiding sugary drinks, is generally recommended, but should be adjusted when consuming foods with a high dietary protein content which leads to a higher daily water requirement.

**13. Nutrition session (after 14 months of intervention)**

In this session, individual V3 food records and nutrition-related blood values are discussed in detail. Moreover, practice-oriented information to achieve the recommended food pattern is collected in the group.

**14. Nutrition session (after 16 months of intervention)**

Eating behavior and differences between signals of hunger, appetite, ravenous appetite, early satiety, long-lasting satiety and fullness are explained in this session. Participants are encouraged to share their eating habits and feelings about this topic. It is emphasized that fast and long-lasting satiety can be achieved by combination of voluminous, water-, fibre-, protein- and moderately fat-containing food products.

**15. Nutrition session (after 18 months of intervention)**

In this session, the topic of eating behaviors is discussed more into detail with focus on emotion-induced eating as well as importance of mindful eating. The complex relationship between food and emotion are discussed based on individual experiences of the participants.

**16. Nutrition session (after 20 months of intervention)**

In this session, pleasurable versus mindless consumption of food is discussed. The ‘rules for pleasure’ are discussed based on a book chapter of *‘Kleine Schule des Genießens’* by Eva Koppenhöfer. The participants are encouraged to pay more attention to pleasurable moments in their everyday life.

**17. Nutrition session (after 22 months of intervention)**

The main topic of this unit is food labeling, especially mandatory disclosers and nutritional information with the aim to train participants to choose suitable foods and to quickly identify and assess the ingredients of food products.

**18. Nutrition session (after 25 months of intervention)**

In this session, individual V4 food records and nutrition-related blood values are discussed in detail. Moreover, practice-oriented information to achieve the recommended food pattern is collected in the group.

**19. Nutrition session (after 28 months of intervention)**

In this session, participants are interviewed on individual realization of the NutriAct food pattern in daily routine. This information exchange is especially aimed at supporting participants who have not implemented this food pattern sufficiently so far. Also, appropriate food products apart from supplied NutriAct products are again presented.

**20. Nutrition session (after 31 months of intervention)**

In this unit, legumes are outlined as valuable sources of plant-based proteins, complex carbohydrates, fibre, vitamins, minerals and phytochemicals. A cooking class is held to provide possibilities of integrating more legumes into meals.

**21. Nutrition session (after 34 months of intervention)**

In this session, participants solve a crossword puzzle with questions referring to important contents of former group sessions as a way to summarize and repeat key information for the intervention group. Then, participants are encouraged to share realized dietary changes and associated perceived effects during the whole study period. For the last time, participants are requested to optimize implementation of NutriAct food pattern until the final phenotyping procedure (V5).

**Overview of nutrition sessions for control group**

**1. Nutrition session (4-6 weeks after V0)**

The aims and the concept of the study are again presented to the participants. The main topic is a detailed description of the food-based dietary recommendations of the DGE for a balanced health-promoting diet. Each participant receives individual food-based and nutrient-based analyses of V0 food records with discussion of results in the group. Special attention is paid to the intake of total fat and subjects are informed about different kinds of fatty acids (SFA, MUFA and PUFA) and their differential effects (e.g. adverse effects of SFA) on blood cholesterol levels. In this context, participants are trained to recognize the amount of total fat and SFA on food labels. Thereby they are advised to consume more low-fat food products. Each participant receives a food pyramid as a non-food gift which illustrates the wholesome diet.

**2. Nutrition session (4-8 weeks after V1)**

Each participant receives individual analyses of V1 food records and the results are discussed using the DGE recommendation for macronutrient composition^3^. Participants also receive the analysis of certain nutrition-related blood parameters (e.g. LDL cholesterol, serum triglycerides). The relation between diet and blood values is explained. Moreover, strategies to change eating habits in line with the DGE recommendations are outlined in detail.

**3. Nutrition session (4-8 weeks after V2)**

Each participant receives individual analyses of V2 food records and of nutrition-related blood values. Main topic of the unit is the long-term change of eating behavior with particular focus on reducing consumption of sweets, sweet bakery products and alcoholic drinks.

**4. Nutrition session (4-8 weeks after V3)**

Individual V3 food records and nutrition-related blood values are again discussed with the participants. The distinction of hunger, appetite, ravenous hunger, early satiety, long-lasting satiety and fullness is also explained in this session. Participants are encouraged to share their eating habits. Advice is given that a combination of voluminous, water-, fibre-, protein- and moderately fat-containing foods provokes a fast and long-lasting satiety. Further, the relationship between food and emotion is discussed based on participants’ individual experiences.

**5. Nutrition session (4-8 weeks after V4)**

Individual V4 food records and nutrition-related blood values are again discussed with the participants. Strategies to implement a healthy diet according to recommendations of the DGE are summarized again. Pleasurable and mindful eating versus mindless consumption of foods is the main topic of the last nutrition session.

**References**

1. Guralnik JM, Simonsick EM, Ferrucci L, et al. A short physical performance battery assessing lower extremity function: association with self-reported disability and prediction of mortality and nursing home admission. *J Gerontol.* 1994;49(2):M85-94.

2. Freese J, Feller S, Harttig U, et al. Development and evaluation of a short 24-h food list as part of a blended dietary assessment strategy in large-scale cohort studies. *Eur J Clin Nutr.* 2014;68(3):324-329.

3. *Referenzwerte für die Nährstoffzufuhr. 2. Auflage, 1. Auflage 2015.* Bonn: Deutsche Gesellschaft für Ernährung, Österreichische Gesellschaft für Ernährung, Schweizerische Gesellschaft für Ernährungsforschung, Schweizerische Vereinigung für Ernährung (Hrsg.).[German]

4. *Die Dreimimensionale Lebensmittelpyramide. In DGE Beratungs-Standards. Bonn, 1. Ergänzungesliferung zur 10. Auflage 2011.* Bonn: Deutsche Gesellschaft für Ernährung (Hrsg.).[German]

**Supplemental Figure and Table Legend**

**Figure S1.** Schedule of nutrition sessions and assessment of food intake of intervention (right) and control group (left). NS, nutrition session. V, visit.

**Table S1.** Food products used in the intervention group.
